# Association Between Sleep Complaints and Homebound Status Among Older Adults: A Cohort Study Using NHATS Data

**DOI:** 10.1093/geroni/igaf122.1032

**Published:** 2025-12-31

**Authors:** Rendong He, Katherine Ornstein, Claire Wang, Junxin Li

**Affiliations:** Johns Hopkins University, Baltimore, Maryland, United States; Johns Hopkins University, Baltimore, Maryland, United States; Johns Hopkins University, Baltimore, Maryland, United States; Johns Hopkins University, Baltimore, Maryland, United States

## Abstract

Homebound status negatively impacts morbidity and quality of life in older adults. While sleep disturbances have been identified as risk factors for various adverse health outcomes, their longitudinal impact on the development of homebound status remains understudied. Using the 2011–2018 National Health and Aging Trends Study (NHATS) data, this longitudinal analysis examined the association between sleep complaints, measured by self-reported difficulties in falling or staying asleep, and the risk of transitioning to homebound status among older adults. To minimize confounding, we excluded individuals with dementia and Activities of Daily Living (ADL) and Instrumental Activities of Daily Living (IADL) impairments, as assessed using relevant NHATS assessment items. Participants who initially reported no sleep complaints at baseline were included. For participants who consistently reported no sleep complaints each year, baseline was defined at the first available wave. For those who developed sleep complaints during follow-up, baseline was redefined at the first occurrence of sleep complaints. Homebound status was defined as never or rarely leaving home. Time-varying Cox proportional hazards models were used to estimate the hazard ratios (HRs) for incident homebound status. Among 1,408 older adults, having two sleep complaints was significantly associated with an increased risk of becoming homebound in the adjusted model controlling for demographics, comorbidities, function, cognition and depression (HR = 2.17, 95% CI [1.15, 4.12]). One sleep complaint was not significantly associated with homebound status in any model. Our findings suggest that addressing sleep complaints may be a valuable strategy for preventing homebound status among older adults.

